# Supplementary material for: The precision of attention controls attraction of population receptive fields
Source: J Vis. 2025 Sep 3;25(11):3. doi: 10.1167/jov.25.11.3 (PMC12410274; doi:10.1167/jov.25.11.3)

Supplementary Figure 1. Attentional precision does not significantly alter *pRF size.* *a)* *pRF size difference (distributed minus focused) for pRFs along the eccentricity range in each visual area. pRFs are binned for both tasks based on an independent pRF mapper. The orange line at the bottom of the plot denotes the stimulus extent of the focused attention task (0-0.3˚ radius), the teal line denotes the stimulus extent of the distributed attention task (0.3-5˚ radius), and the dashed vertical line the boundary between the two tasks. The dashed horizontal line is at 0, representing no difference between focused and distributed attention. Each individual point is an average of all participants within each eccentricity bin. The solid lines are an exponential function fit to the data. The shaded area denotes a bootstrapped 95% confidence interval of the exponential curve fit. All error bars are SEM.* ***b)*** *pRF size difference (distributed minus focused) across the visual hierarchy for pRFs falling within the distributed color task stimulus (0.3-5˚ radius). Values per participant are shown in the scatter plot nested within each bar, group averages in bar plots, and error bars are SEM.*

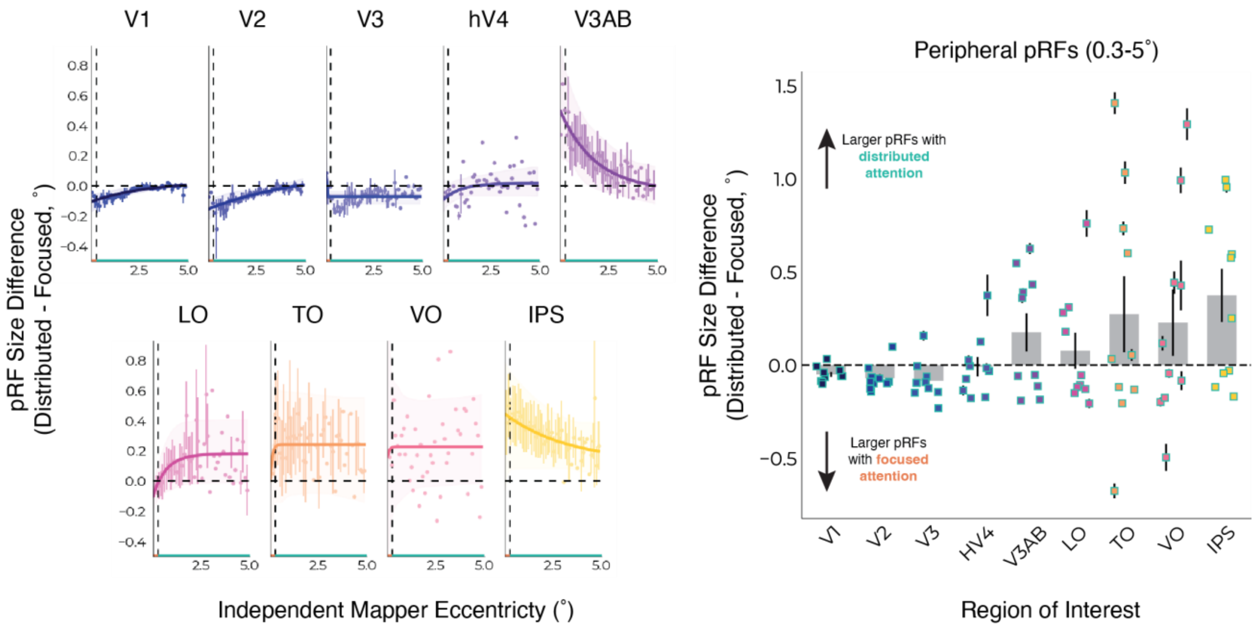

Supplement: Supplement 1 [file jovi-25-11-3_s001.pdf]
