# Supplementary figures and images for: The precision of attention controls attraction of population receptive fields
Source: J Vis. 2025 Sep 3;25(11):3. doi: 10.1167/jov.25.11.3 (PMC12410274; doi:10.1167/jov.25.11.3)

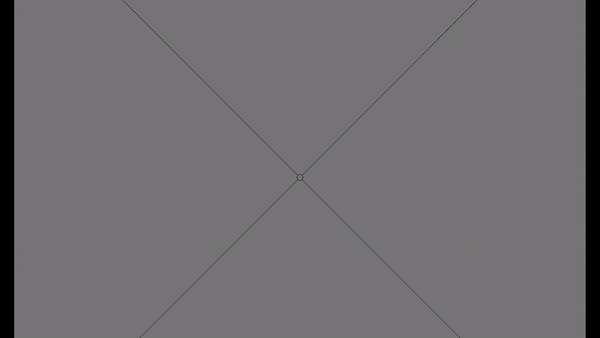

Supplement: Supplement 2 [file jovi-25-11-3_s002.gif]
